# ERRATUM: Quality of Care Scale in Brazilian Portuguese: the perspective of people with disabilities

**DOI:** 10.1590/S0034-8910.2014048005056err

**Published:** 2015-09-29

In the article: “Quality of Care Scale in Brazilian Portuguese: the perspective of people with disabilities” publicado no periodico “Revista de Saúde Pública”, volume 48(4) de 2014, em Annex, Item 9.

Where it reads:

“Does a lack of services where you live limit the care and support you get? *For example, if there is a lack of facilities, staff, or money for medicines or other treatments*.”

It should read:

“Do you get the help you need to live in your home? *For example, help with washing, dressing, cooking, cleaning, etc*.”

